# Metachronous multicentric giant cell tumor of bone treated with denosumab and mega-prosthesis revision: a rare case report

**DOI:** 10.3389/fonc.2025.1651271

**Published:** 2025-12-10

**Authors:** Huaitai Lin, Chanping Yang, Jinhao Zhu, Chenyu Yang, Zijian Xu, Zejin Wu, Xinjia Wang, Weidong Wang

**Affiliations:** 1 Orthopedics Department, Cancer Hospital of Shantou University Medical College, Shantou, Guangdong, China; 2 Operating Room, Cancer Hospital of Shantou University Medical College, Shantou, Guangdong, China

**Keywords:** multicentric giant cell tumor, metachronous multicentric GCTB, mega-prosthesis, denosumab, curettage

## Abstract

**Introduction:**

Multicentric giant cell tumor of bone is extremely rare, accounting for less than 1% of all giant cell tumor of bone (GCTB) cases. Metachronous multicentric GCTB is even rarer.

**Case presentation:**

We present a rare case of metachronous multicentric giant cell tumor of bone (GCTB) in a 27-year-old male, in whom three distinct lesions developed sequentially over a 10-year period: first in the right distal femur, followed by the proximal femur, and later the proximal tibia. To address progressive disease and prosthetic complications, the patient underwent revision of the mega-prosthesis combined with adjuvant denosumab therapy, followed by curettage of the subsequently emerging tibial lesion.

**Conclusion:**

This case illustrates the sequential development of multiple GCTB lesions and the importance of combined surgical reconstruction and denosumab therapy in long-term management.

## Introduction

Giant cell tumor of bone (GCTB) is a benign but locally aggressive primary bone neoplasm, accounting for 4–5% of all primary bone tumors. It most commonly occurs in the third to fifth decades of life and affects both males and females, with a slight female predominance (1, 2). Multicentric giant cell tumor of bone is rare, representing less than 1% of all GCTB cases, and typically presents as multiple synchronous or metachronous lesions in different bones (3–5). “Synchronous” was defined as multiple tumors discovered at the initial presentation or when a second tumor was diagnosed within 6 months of the first, whereas “metachronous” referred to cases in which the second tumor developed more than 6 months after the first lesion (2). Patients with multicentric GCTB usually present with two or three lesions. Reported series indicate that multicentric GCTB tends to occur at a younger age than solitary GCTB, with a mean age of 22.5 years. Although multicentric GCTBs, like their solitary counterparts, commonly affect the long bones around the knee, they more frequently occur at atypical sites such as the small bones of the hands and feet, as well as at metaphyseal or metadiaphyseal regions of the bone (6). Other polyostotic skeletal lesions are relatively common, and differential diagnoses should be carefully considered. Histopathological confirmation is sufficient for a definitive diagnosis in most cases. However, brown tumors may exhibit histological features similar to GCT; therefore, hyperparathyroidism must be excluded through a parathyroid hormone (PTH) assay.

Due to the rarity of multicentric GCTB, its biological behavior and optimal management remain poorly understood.

We present a patient who was 17 years old at the initial presentation and had developed three lesions by the age of 27.

## Case presentation

A 27-year-old male patient was admitted to our department in April 2024 due to the identification of a new lesion in the right lateral proximal tibia, which was diagnosed as a giant cell tumor.

In April 2014, he was diagnosed for the first time with a giant cell tumor of the right distal femur at another hospital and underwent curettage and bone grafting. In April 2017, he developed a second lesion in the right proximal femur and underwent resection of the proximal femoral tumor segment followed by tumor-type hemiarthroplasty. In October 2023, he underwent acetabular revision surgery due to acetabular wear.

Surgical treatment was recommended for the tibial lesion; however, due to psychological fear of surgery, the patient was treated with denosumab (120 mg on Day 1, Day 8, and Day 15 of the first month, followed by one injection every 28 days).

In June 2024, the patient underwent right hip mega-prosthesis revision surgery with allogeneic bone reconstruction and internal fixation due to prosthesis failure. During this hospitalization, follow-up imaging revealed significant reactive sclerosis and cortical thickening of the tibial lesion.

In April 2025, curettage of the right proximal tibial lesion was performed, followed by bone filling using polymethylmethacrylate (PMMA). Postoperative pathological examination revealed no residual tumor.

**Figure 1:**
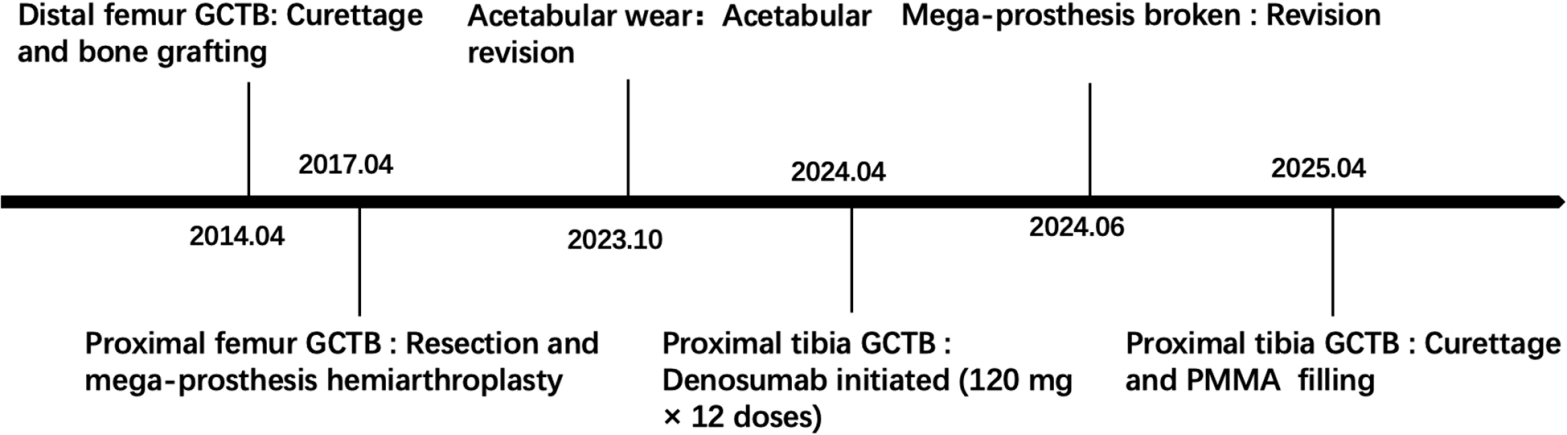
Clear and effective timeline.

A concise summary of the diagnostic and therapeutic timeline is provided in **Figure 1**.

### Denosumab therapy

**Figure 2:**
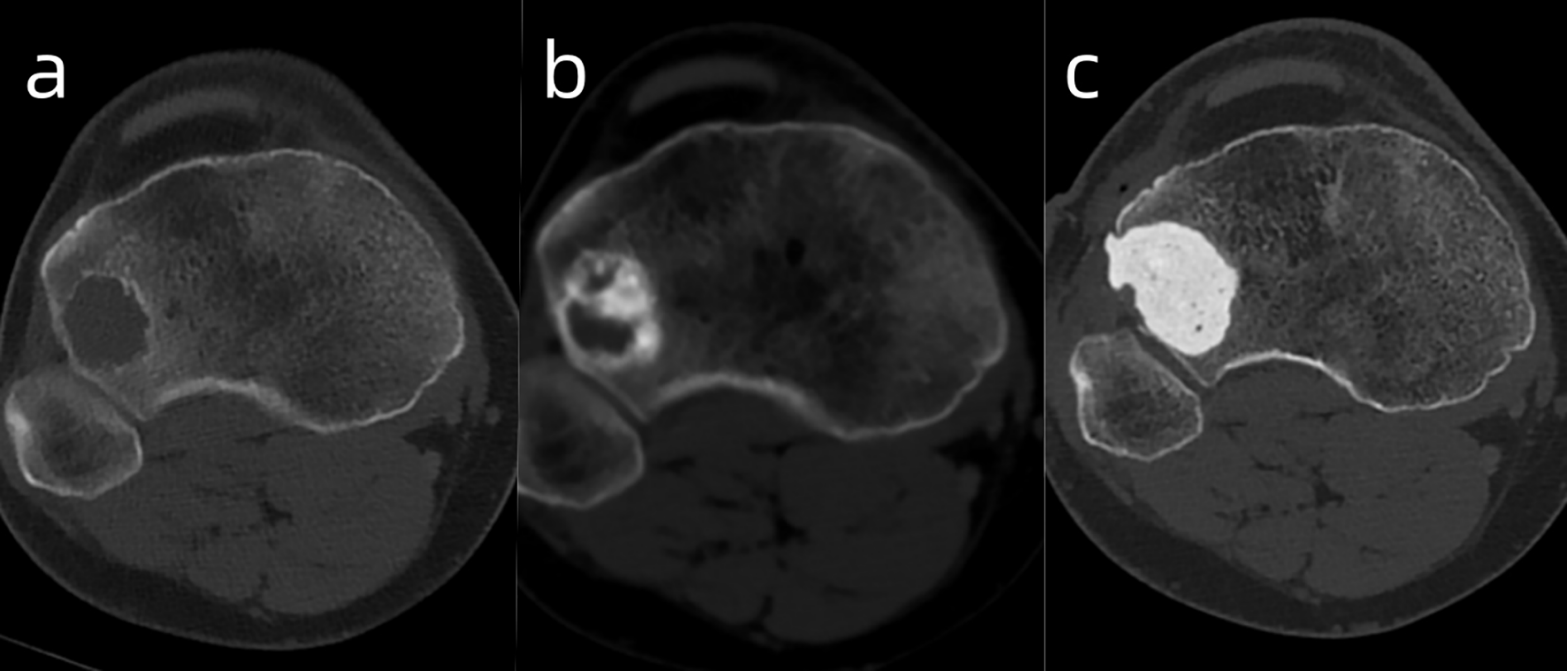
The images illustrate the GCT of the right proximal tibia before **(A)**, after denosumab [**(B)** - showing reactive sclerosis and cortical thickening], and after curettage/PMMA filling **(C)**.

The patient was admitted to our hospital on April 11, 2024. A CT scan revealed a new giant cell tumor of the right proximal tibia (The third site)(**Figure 2a**). Surgical treatment was recommended; however, due to psychological fear of surgery, the patient was instead treated with denosumab (120 mg on Day 1, Day 8, and Day 15 of the first month, followed by one injection every 28 days, for a total of 12 injections).

**Figure 3:**
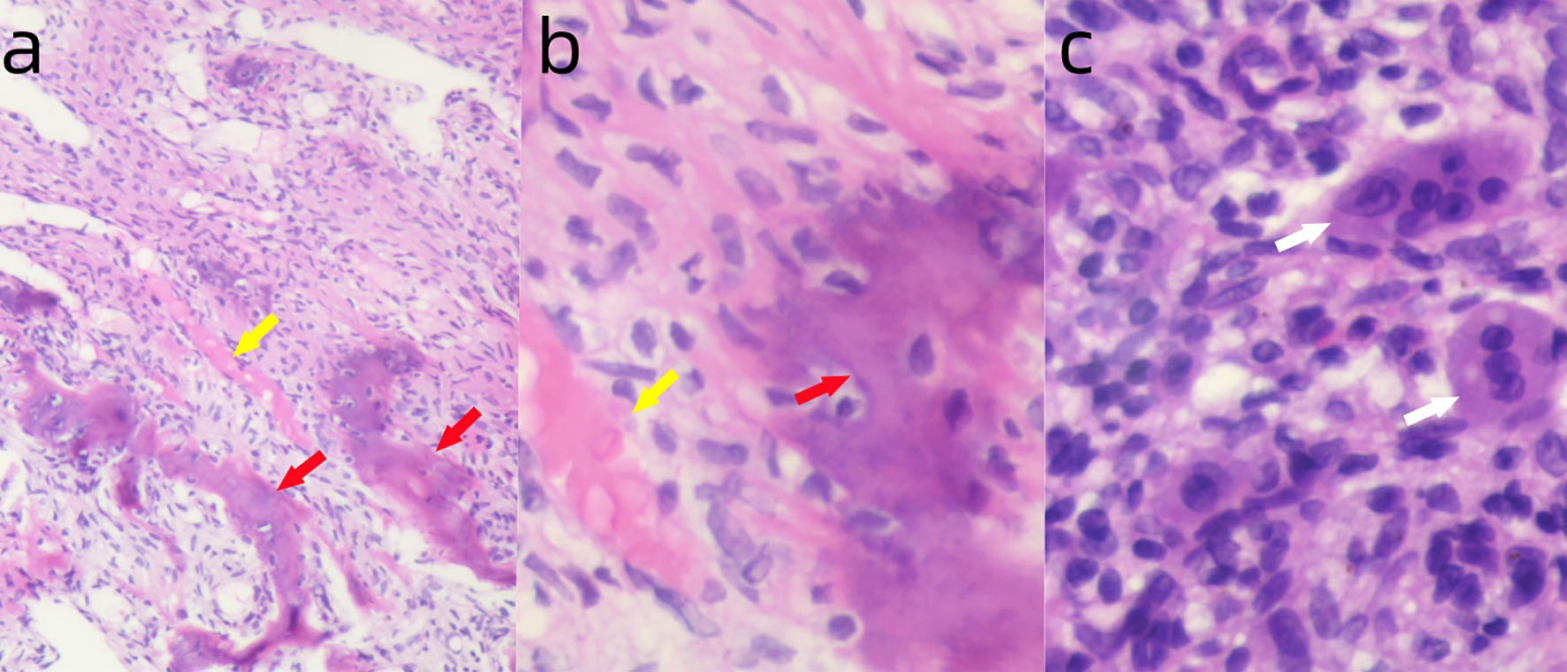
**(a)** Postoperative pathology showing fibrous tissue hyperplasia (yellow arrow) and new woven bone formation (red arrow). H&E ×100. **(b)** Higher magnification showing fibrous tissue hyperplasia (yellow arrow) and new woven bone formation (red arrow). H&E ×400. **(c)** Small foci of multinucleated giant cell infiltration (white arrow). H&E ×400.

Follow-up examinations after treatment showed marked reactive sclerosis and cortical thickening within the tumor (**Figure 2b**). The patient was informed that only a portion of the lesion had become sclerotic and hypertrophic, while the remaining areas showed no significant change, as denosumab has no direct effect on tumor stromal cells, which may lead to tumor recurrence or sarcomatous transformation. Moreover, long-term administration of this agent may cause serious adverse effects, such as osteonecrosis of the jaw, whereas discontinuation of the drug may result in accelerated tumor progression. Considering these potential risks, the patient ultimately consented to surgical intervention. On April 21, 2025, the patient underwent curettage of the lesion in the right proximal tibia followed by filling with polymethylmethacrylate (PMMA)(**Figure 2c**). The postoperative pathology showed the intended therapeutic effect of denosumab, characterized by prominent fibrous tissue hyperplasia and extensive formation of new woven bone. Only scattered small foci of multinucleated giant cells remain, indicating a marked reduction in tumor cellularity and stromal activity (**Figure 3**).

### Mega-prosthesis revision

**Figure 4:**
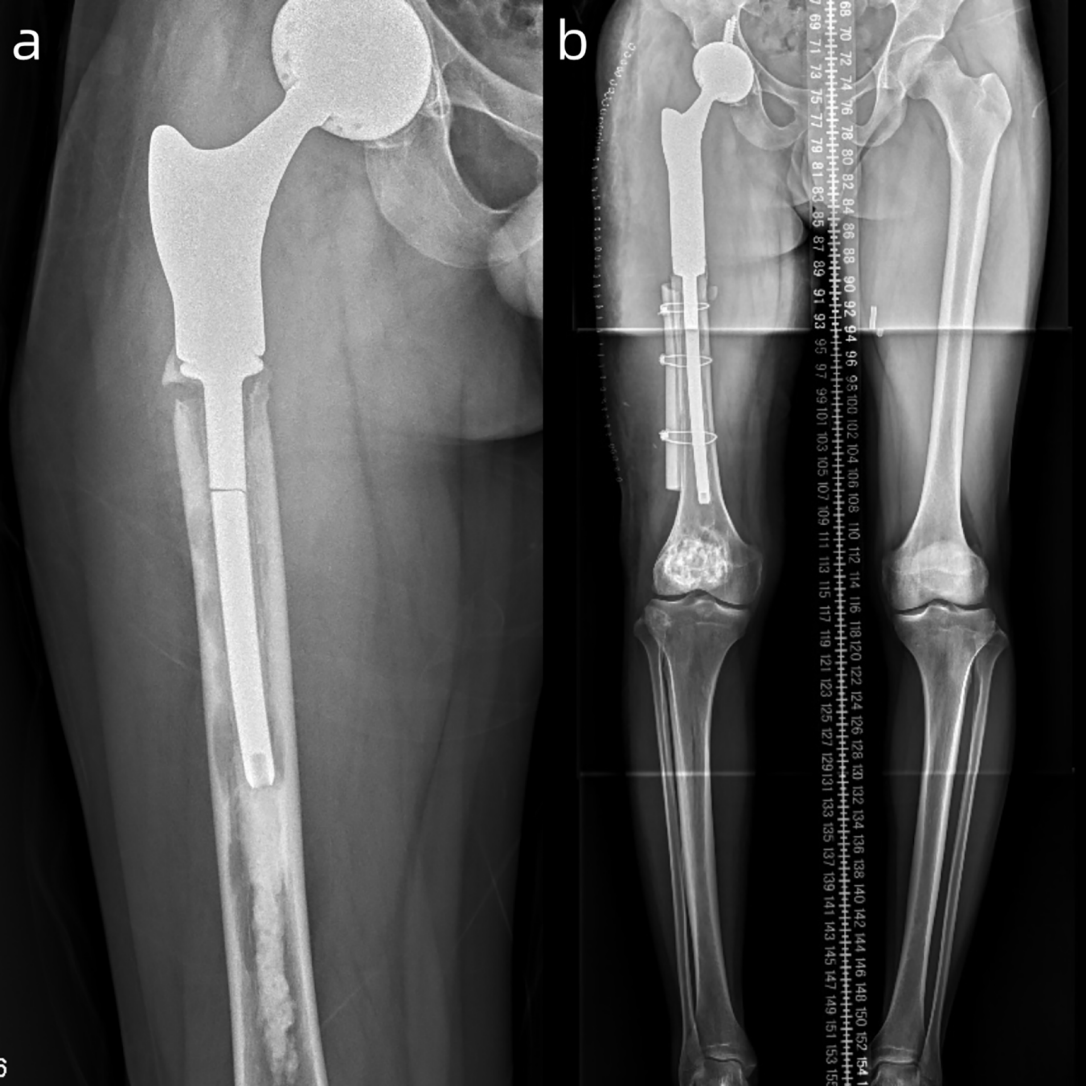
**(a)** X-ray clearly demonstrates the broken extended stem of the right femoral prosthesis. **(b)** Full-length X-ray confirms the successful revision with the prosthesis in a good position and equal limb lengths.

The patient was admitted to our department on June 7, 2024, with complaints of right thigh pain and abnormal movement. X-ray and CT showed that the extended stem of the right femoral prosthesis was broken in the medullary cavity (**Figure 4a**). On June 18, 2024, he underwent right hip mega-prosthesis revision surgery with allogeneic bone reconstruction and internal fixation. The postoperative full-length X-ray of the lower limbs showed that the prosthesis was well positioned and that both lower limbs were of equal length (**Figure 4b**). The patient walked normally after 3 months of the operation.

## Discussion

Giant cell tumor of bone (GCTB) is a locally aggressive benign bone tumor, accounting for 4–5% of all primary bone tumors. It most commonly occurs in the third to fifth decades of life and affects both males and females, with a slight female predominance (1, 2). GCTB is typically a solitary lesion that most commonly occurs in the distal femur, proximal tibia, distal radius, and proximal humerus. It predominantly involves the epiphysis and may extend into the adjacent metaphysis.

Multicentric GCTB is rare, representing less than 1% of all GCTB cases, and typically presents as multiple synchronous or metachronous lesions in different bones (3–5). Hoch et al. (2) classified tumors as synchronous when multiple lesions were identified at the initial presentation, or when a second tumor was diagnosed within six months of the first. In contrast, tumors were classified as metachronous if the second lesion developed more than six months after the first. A literature review of reported cases of multicentric giant cell tumor worldwide, conducted by G. Trovarelli et al. (7) in 2022, revealed a total of 106 diagnosed cases. Patients with multicentric GCTB tend to be younger and show a slight female predominance compared to those with solitary GCTB. In fact, more than 80% of patients with solitary GCTB are older than 25 years, whereas approximately 70% of patients with multicentric GCTB in our review were younger than 25 years. In metachronous cases, the mean interval between the first and subsequent lesions was seven years (range: six months to 27 years). The mean number of affected sites was 3.5 (range: 2 to 12), with four or more tumors identified in 30% of patients. Lesion involvement was ipsilateral in 47% of cases, on the right side in 43%, and bilateral in 33%. Axial skeleton involvement was observed in 10% of cases. Multicentric GCTB is biologically more aggressive than solitary GCTB. Approximately 60% of patients with multicentric GCT experience local recurrence in at least one operated lesion, and 13% develop metastases. In contrast, local recurrence and metastasis occur in less than 10% and 2% of patients with solitary GCTB, respectively (4). As with solitary giant cell tumor of bone, surgical resection remains the preferred treatment approach, most commonly including curettage, wide resection, and, in rare cases, amputation. Other treatments include radiotherapy, cryotherapy, phenol, or embolization.

Pathogenesis of giant cell tumor of bone (GCTB): Bone morphogenesis, remodeling, and resorption are primarily regulated by osteoclasts; therefore, osteoclasts play a central role in these processes (8). Osteoprotegerin (OPG) serves as a negative regulator of osteoclastogenesis, whereas the OPG ligand/RANKL/TRANCE pathway plays a critical role in promoting osteoclast differentiation by activating genes involved in this process. RANKL/TRANCE stimulates osteoclastogenesis through the RANK receptor by activating the NF-κB signaling pathway via TRAF6. Once activated, NF-κB translocates to the nucleus and induces the expression of osteoclastogenic genes. OPG inhibits osteoclastogenesis by binding to RANKL and preventing its interaction with RANK. Notably, RANKL is essential for osteoclast survival and plays a crucial role in the pathogenesis of GCTB (9). In GCTB, neoplastic stromal cells overexpress receptor activator of nuclear factor kappa B ligand (RANKL), which binds to its receptor RANK on osteoclast precursors and promotes their differentiation into mature, multinucleated osteoclast-like giant cells. These giant cells mediate excessive bone resorption, leading to the locally aggressive and osteolytic nature of the tumor. The neoplastic stromal cells, rather than the giant cells, are considered the true neoplastic component of GCTB, as they drive the recruitment and activation of osteoclasts through the RANK–RANKL signaling pathway (10). By binding to RANKL with high affinity, denosumab prevents its interaction with the RANK receptor on osteoclast precursors, thereby inhibiting the formation of osteoclast-like giant cells and suppressing tumor-induced osteolysis. It has various clinical applications, including the treatment of osteoporosis in postmenopausal women at high risk of fracture, the management of bone metastases associated with solid tumors, and the treatment of advanced or inoperable giant cell tumor (11–13). Histologically, GCTB is generally composed of three main types of cells: mononuclear tumor-like stromal cells with an osteoblastic precursor phenotype, mononuclear histiocytes, and osteoclast-like multinucleated giant cells (14). GCTB contains a large number of giant cells, approximately 92% of which harbor the H3F3A mutation (15). Although denosumab effectively reduces the number of osteoclast-like giant cells, the H3F3A*-*mutated neoplastic stromal cells persist after treatment and undergo significant histological changes in response, which may contribute to tumor recurrence (16).

Systemic treatment with denosumab can be beneficial in cases involving multiple sites. In patients with GCT, denosumab provides pain relief, facilitates surgical procedures by reducing tumor vascularity and intraoperative bleeding, and promotes tumor ossification and clear demarcation from surrounding soft tissues, thereby reducing the risk of neurovascular injury (17–22). Denosumab has been used as adjuvant therapy before surgery or as palliative treatment for unresectable and advanced giant cell tumor of bone. Although there are reports on the use of denosumab in multicentric giant cell tumor of bone replacing surgical treatment, a clear consensus regarding its efficacy and treatment guidelines has yet to be established (23, 24).

Tyrosine kinases (TKs) play essential roles in cellular signaling, angiogenesis, and cell cycle regulation. Given their frequent dysregulation in cancer, tyrosine kinase inhibitors (TKIs) have become important therapeutic agents in oncology (25–27). In giant cell tumor of bone (GCTB), genes associated with bone vascular formation (OPG) and neoangiogenesis (FLT1) exhibit opposite expression patterns, with OPG significantly downregulated and FLT1 upregulated. These findings suggest a potential therapeutic role for novel antiangiogenic agents, such as the multitarget tyrosine kinase inhibitor lenvatinib, in the management of these lesions. Moreover, recent evidence indicates that the combination of denosumab and lenvatinib may enhance therapeutic efficacy, as angiogenesis is a major hallmark of tumorigenesis and VEGFR (a target of lenvatinib) participates in RANKL-induced osteoclastogenesis. This combined approach may also reduce intraoperative bleeding by decreasing tumor vascularity (28).Furthermore, previous studies have shown that denosumab inhibits osteoclast-like giant cells and modulates receptor tyrosine kinase signaling in GCTB, while sunitinib, a PDGFR inhibitor, significantly reduces the viability of tumor stromal cells by inhibiting PDGFR signaling, thereby increasing osteoprotegerin production in osteoblastic/stromal cells and attenuating RANKL/RANK signaling. Collectively, these findings indicate that combined therapy targeting both osteoclastic and stromal components represents a promising precision treatment strategy for locally advanced or metastatic GCTB (29–31).

With advancements in comprehensive limb-salvage techniques, mega-prosthesis is increasingly utilized following tumor resection involving adjacent joints. However, these prostheses are frequently associated with complications. Postoperative complications can be classified as mechanical or non-mechanical, as well as early or late, depending on the time of onset and underlying etiology. Non-mechanical complications, such as periprosthetic infections, are particularly serious and require tailored management strategies (32, 33).

Mechanical complications may include (34):

- Aseptic loosening of components (particularly intramedullary stems),
- Implant fracture,
- Disconnection of modular components,
- Hip dislocation following proximal or total femoral replacement,
- Wear of components (especially polyethylene parts),
- Periprosthetic fractures.

Overall, the failure rate of primary procedures is approximately 29%. When analyzed by anatomical location, proximal femoral replacements demonstrate a failure rate of around 20%. Time to failure varied significantly depending on the anatomical location of the mega-prosthesis. The overall mean time to failure was 47 months. The shortest mean time to failure—10.9 months—was observed in distal humeral replacements, while the longest, 53 months, was noted in proximal humeral replacements. Proximal tibial and distal femoral replacements demonstrated similar failure intervals (34).

This patient experienced both femoral head component wear at 114 months and intramedullary stem implant fracture at 122 months. During the operation, the intramedullary broken end of the prosthesis and the surrounding bone cement could not be removed using standard techniques. Therefore, a cortical window was created in the femur at the distal end of the prosthesis, allowing successful extraction of both the broken implant and cement. A cemented extended femoral stem was then implanted. Given the risk of postoperative fracture at the osteotomy site, allogeneic bone plates and cables were applied bilaterally to reinforce the femur and reduce the risk of stress-related fractures.

## Conclusion

In summary, metachronous multicentric GCTB is rare and tends to occur in younger patients. A thorough history and physical examination should be conducted in all young patients presenting with GCTB to identify potential additional lesion sites. In our case, the patient had a multicentric metachronous GCTB requiring multiple surgeries for local control. Regular follow-up after prosthetic reconstruction is essential to detect and manage complications promptly. Any symptomatic bone lesion should undergo further radiological evaluation due to the possibility of multicentric involvement. Additionally, hyperparathyroidism should be excluded. In patients with larger tumors, denosumab can be used as neoadjuvant therapy to reduce intraoperative bleeding and minimize collateral damage. For unresectable tumors, denosumab may serve as maintenance therapy to shrink the lesion and relieve pain. In addition, other therapeutic approaches, such as tyrosine kinase inhibitors, have also demonstrated certain efficacy.

## Data Availability

The original contributions presented in the study are included in the article/supplementary material. Further inquiries can be directed to the corresponding author.
